# Cross-Reactivity of 15 JEV Antigen-Directed Rabbit Antisera with Three Clinically Important JEV Serogroup Members: WNV, MVEV, and SLEV

**DOI:** 10.3390/v18080873

**Published:** 2026-08-10

**Authors:** Sang-Im Yun, Young-Min Lee

**Affiliations:** Department of Animal, Dairy, and Veterinary Sciences, College of Agriculture and Natural Resources, Utah State University, Logan, UT 84322, USA

**Keywords:** Japanese encephalitis virus, West Nile virus, Murray Valley encephalitis virus, St. Louis encephalitis virus, serogroup, serocomplex, antigenic complex, cross-reactivity

## Abstract

Japanese encephalitis virus (JEV), the prototype member of the JEV serogroup within the genus *Orthoflavivirus* (family *Flaviviridae*), is closely related to West Nile virus (WNV), Murray Valley encephalitis virus (MVEV), and St. Louis encephalitis virus (SLEV). To characterize antigenic cross-reactivity within this group, we evaluated 15 region-specific rabbit antisera, previously generated against nearly the entire JEV protein-coding region, by immunoblotting whole-cell lysates from BHK-21 cells infected with WNV, MVEV, or SLEV, with JEV included as a reference. Six antisera (α-E^N-term^, α-NS2B, α-NS3^N-term^, α-NS3^C-term^, α-NS5^N-term^, and α-NS5^C-term^) robustly recognized homologous proteins across all three viruses. The remaining nine antisera displayed lineage-restricted or virus-specific reactivity: (*a*) α-C cross-reacted strongly with WNV and MVEV but weakly with SLEV; (*b*) α-M, α-NS1, α-NS4A, and α-NS4B^C-term^ cross-reacted with WNV and MVEV but not SLEV; (*c*) α-E^C-term^ and α-NS1′ cross-reacted only with WNV; (*d*) α-Pr cross-reacted exclusively with MVEV; and (*e*) α-NS4B^N-term^ showed no detectable cross-reactivity under the experimental conditions used. Notably, α-NS1 and α-NS1′ detected heat-labile multimers of NS1 and NS1′. These serological patterns mirror the established phylogeny of the JEV serogroup, with JEV clustering most closely with MVEV, followed by WNV and then SLEV. Together, these findings provide a comprehensive cross-reactivity map of JEV antigen-directed antisera and establish a practical framework for dissecting antigenic relationships among JEV serogroup members. These results enhance our understanding of orthoflavivirus antigenic evolution and support the future development of improved diagnostics, broad-acting vaccines, and experimental reagents for emerging and re-emerging encephalitic orthoflaviviruses.

## 1. Introduction

Japanese encephalitis virus (JEV) is the prototypical member of the JEV serogroup (also referred to as the serocomplex or antigenic complex) within the genus *Orthoflavivirus* of the family *Flaviviridae* [1]. This serogroup includes several clinically important orthoflaviviruses, most notably West Nile virus (WNV), Murray Valley encephalitis virus (MVEV), and St. Louis encephalitis virus (SLEV), as well as additional viruses of more limited clinical relevance, such as Usutu virus (USUV), Koutango virus (KOUV), Yaoundé virus (YAOV), and Cacipacoré virus (CPCV) [2,3]. These zoonotic arboviruses circulate in complex transmission cycles involving birds and other vertebrate hosts, with *Culex* or *Aedes* mosquitoes serving as primary vectors. Humans are incidental, dead-end hosts following spillover infection [4,5]. Phylogenetically, JEV serogroup members form a closely related clade that has diverged into distinct lineages reflecting their evolutionary histories [6,7]. Among mosquito-borne orthoflaviviruses, the JEV serogroup is one of seven established serogroups, alongside Aroa, dengue, Kokobera, Ntaya, Spondweni, and yellow fever viruses. Dengue and yellow fever viruses, in particular, underscore the broader public health significance of orthoflaviviruses as globally important emerging and re-emerging pathogens [8,9].

Orthoflaviviruses cause a wide spectrum of clinical outcomes in humans, ranging from mild febrile illness to severe neurological or hemorrhagic disease [10]. The four most medically important members of the JEV serogroup, namely JEV, WNV, MVEV, and SLEV, are neurotropic viruses capable of causing life-threatening encephalitis [5,11]. JEV remains a leading cause of viral encephalitis in the Asia–Pacific region, with high morbidity and mortality among children [12,13]. WNV, now widely distributed across Africa, the Middle East, Europe, Asia, Australia, and the Americas, can cause neuroinvasive disease such as meningitis and encephalitis, particularly in older or immunocompromised individuals [14,15]. MVEV, endemic to New Guinea and Australia, produces sporadic outbreaks with substantial case fatality [16,17], whereas SLEV, circulating in the Americas, has caused sporadic encephalitis outbreaks [18,19]. Across all four viruses, a significant proportion of survivors experience long-term neurological sequelae [20,21]. Collectively, these antigenically related encephalitic orthoflaviviruses pose major global health threats, particularly in tropical and subtropical regions with dense human populations [5,22]. In New Guinea and Australia, the co-circulation of JEV, WNV, and MVEV complicates differential diagnosis, surveillance, and outbreak response [23]. In the United States, where WNV and SLEV are endemic, the potential introduction of JEV represents an additional public health concern requiring vigilant monitoring and preparedness [24,25].

Orthoflaviviruses are small enveloped viruses with an approximately 11 kb positive-sense RNA genome [26], capped at the 5′ end but lacking a poly(A) tail at the 3′ end [27,28]. The genome encodes a single open reading frame (ORF) flanked by noncoding regions that regulate viral genome replication, gene expression, transmission, pathogenicity, and innate immune responses [29,30,31,32,33]. Translation of the ORF yields a polyprotein that is cleaved by viral and host proteases into ten proteins: C, prM, E, NS1, NS2A, NS2B, NS3, NS4A, NS4B, and NS5 [34,35]. The structural proteins (C, prM, and E) mediate virion assembly and host cell entry [36,37,38], whereas the nonstructural proteins (NS1 to NS5) coordinate viral RNA replication, polyprotein processing, particle assembly, and modulation of host innate immunity [39,40,41]. In JEV serogroup viruses, a −1 ribosomal frameshift at codons 8 to 9 of NS2A produces an NS1 isoform (NS1′) containing 52 additional amino acids at the C-terminus [42,43]. NS1 and NS1′ form heat-labile multimers involved in viral replication, pathogenesis, and immune modulation [44,45]. NS3 and NS5 are multifunctional enzymes: NS3 contains an N-terminal NS2B-dependent protease domain and a C-terminal helicase/NTPase/RTPase domain [46,47,48], whereas NS5 contains an N-terminal methyltransferase/guanylyltransferase domain and a C-terminal RNA-dependent RNA polymerase domain [49,50]. During viral morphogenesis, prM is cleaved by the host protease furin to generate the mature M protein [51,52]. Although the viral proteins and their functions are relatively well characterized [53,54], the high sequence conservation among orthoflaviviruses, particularly JEV, WNV, MVEV, and SLEV, results in extensive serological cross-reactivity, complicating differential diagnostics and comparative studies [3,4,55].

In this study, we used a panel of 15 region-specific rabbit antisera, previously generated to span nearly the entire JEV protein-coding region [56], to systematically and comprehensively examine their cross-reactivity with WNV, MVEV, and SLEV by immunoblotting infected cell lysates, with JEV included as a reference. The resulting cross-reactivity map provides a framework for defining serological relationships within the JEV serogroup in the context of their phylogenetic relatedness and serves as a valuable resource for comparative virology and the study of these medically important pathogens.

## 2. Materials and Methods

### 2.1. Cells

BHK-21 cells were maintained in α-Minimum Essential Medium (α-MEM) supplemented with 10% fetal bovine serum, 2 mM L-glutamine, 1× MEM vitamin solution, and 1× penicillin–streptomycin (100 U/mL penicillin and 100 μg/mL streptomycin), as described previously [57]. Cultures were incubated at 37 °C in a humidified atmosphere containing 5% CO_2_. All cell-culture reagents were obtained from Gibco (Carlsbad, CA, USA).

### 2.2. Viruses

Four JEV-serogroup viruses were used in this study: (*a*) JEV strain CNU/LP2, derived from a full-length infectious cDNA clone [57]; (*b*) WNV strain B956, obtained from BEI Resources (Manassas, VA, USA); (*c*) MVEV strain H102/94, provided by the Health Protection Agency (Porton Down, Salisbury, UK); and (*d*) SLEV strain H109/92, also provided by the Health Protection Agency.

### 2.3. Antisera

A panel of 15 region-specific polyclonal rabbit antisera recognizing nearly all JEV gene products was previously generated and characterized [56]. Fourteen antisera were produced by immunizing rabbits with recombinant glutathione S-transferase (GST) fusion proteins expressed in *Escherichia coli*. The remaining antiserum, directed against NS2B, was generated using a synthetic peptide conjugated to keyhole limpet hemocyanin (KLH). Additional methodological details are provided in Section 3.

### 2.4. Immunoblotting

Immunoblotting was performed as described previously [56]. Briefly, cells were lysed in 1× sample buffer (80 mM Tris-HCl [pH 6.8], 2% sodium dodecyl sulfate [SDS], 10% glycerol, 100 mM dithiothreitol, and 0.2% bromophenol blue). Lysates were either boiled for 10 min or left unboiled before separation by SDS–PAGE and transfer to polyvinylidene difluoride membranes. Membranes were blocked for 1 h with 5% nonfat dry milk (NFDM) in wash buffer, washed three times for 10 min each, and incubated for 2 h with one of the 15 region-specific JEV rabbit antisera diluted in wash buffer containing 0.5% NFDM. Antisera were used at the following dilutions: α-C (1:4000), α-Pr (1:4000), α-M (1:1000), α-E^N-term^ (1:4000), α-E^C-term^ (1:100), α-NS1 (1:4000), α-NS1′ (1:1000), α-NS2B (1:1000), α-NS3^N-term^ (1:1000), α-NS3^C-term^ (1:1000), α-NS4A (1:1000), α-NS4B^N-term^ (1:500), α-NS4B^C-term^ (1:500), α-NS5^N-term^ (1:1000), and α-NS5^C-term^ (1:1000). As a loading control, a rabbit polyclonal anti-glyceraldehyde-3-phosphate dehydrogenase (α-GAPDH) antiserum (LabFrontier Co., Seoul, Republic of Korea) was used at a dilution of 1:5000. After three additional washes, membranes were incubated for 2 h with alkaline phosphatase (AP)–conjugated goat α-rabbit IgG (Jackson ImmunoResearch, West Grove, PA, USA) diluted 1:5000 in wash buffer containing 0.5% NFDM. Blots were developed using a chromogenic substrate solution of 5-bromo-4-chloro-3-indolyl phosphate (BCIP) and nitroblue tetrazolium (NBT). All immunoblots are representative of at least two independent experiments with consistent outcomes. Immunoblot band intensities were quantified by densitometric analysis using ImageJ (Version 1.51; National Institutes of Health, Bethesda, MD, USA). For each sample, the intensity of each viral-protein band was divided by the intensity of the corresponding GAPDH band. The resulting viral-protein/GAPDH ratio was then normalized to the ratio obtained for the JEV control sample, which was assigned a value of 1.0.

### 2.5. RNA Pseudoknot Prediction

RNA pseudoknot structures spanning nucleotides 3551–3628 of the JEV CNU/LP2 genome were predicted using the IPknot algorithm [58] via the Rtips web server (http://ws.sato-lab.org/rtips/; accessed on 4 January 2026).

### 2.6. Sequence and Phylogenetic Analysis

Multiple nucleotide sequence alignments were generated using ClustalX [59]. Phylogenetic trees were constructed using the neighbor-joining method [60], and branch support was assessed by bootstrap analysis with 1000 replicates [61]. Trees were visualized using TreeView [62].

## 3. Results

### 3.1. Overview of 15 Region-Specific Polyclonal Rabbit Antisera Raised Against JEV Proteins

**Figure 1:**
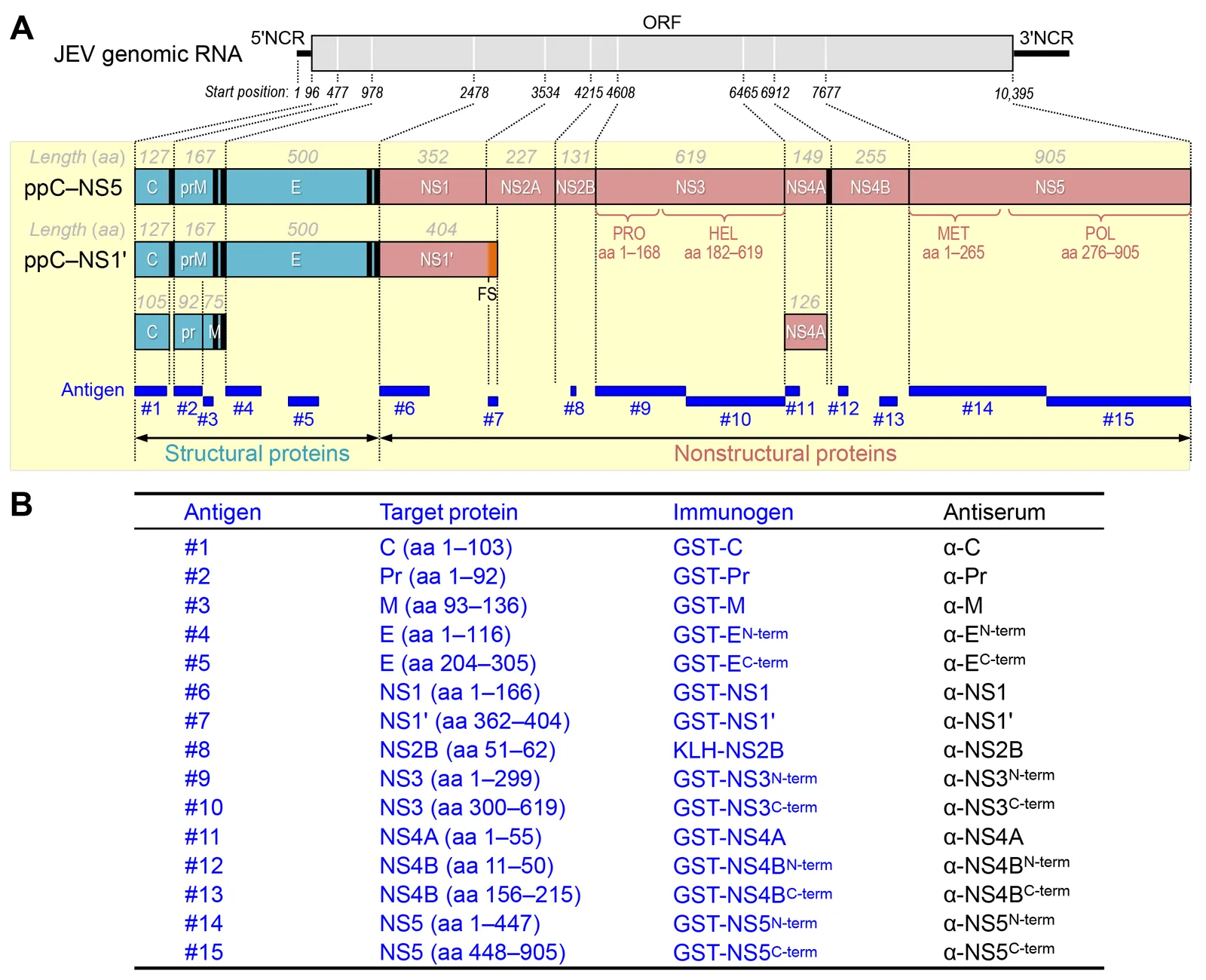
Summary of 15 region-specific rabbit antisera targeting JEV proteins. (**A**) Schematic representation of the 15 JEV antigenic regions used for antiserum production. The top panel shows the genomic organization of JEV strain CNU/LP2, which contains a single long open reading frame (ORF) flanked by 5′ and 3′ noncoding regions (NCRs). Below the genome are two polyprotein precursors, ppC–NS5 and ppC–NS1′, the latter generated by a −1 ribosomal frameshift (FS) at the beginning of NS2A. Proteolytic processing yields 11 functional proteins: C, prM, E, NS1/NS1′, NS2A, NS2B, NS3, NS4A, NS4B, and NS5. NS3 contains an N-terminal protease (PRO) domain and a C-terminal helicase/NTPase/RTPase (HEL) domain, whereas NS5 contains an N-terminal methyltransferase/guanylyltransferase (MET) domain and a C-terminal RNA-dependent RNA polymerase (POL) domain. During the late stage of viral replication, prM is cleaved into the pr fragment and the mature M protein. Protein lengths (amino acids, aa) are indicated above each protein. Horizontal blue bars denote the positions of the 15 antigenic regions used to generate the corresponding antisera. (**B**) Brief description of the 15 antigens, their target proteins (with antigenic regions indicated in parentheses), the immunogens used for rabbit immunization, and the resulting antisera. Fourteen antisera were generated using recombinant GST fusion proteins, and one antiserum (α-NS2B) was generated using a KLH-conjugated synthetic peptide.

To evaluate antigenic cross-reactivity between JEV and three closely related, medically important JEV-serogroup members (WNV, MVEV, and SLEV), we used a panel of 15 well-characterized, region-specific rabbit antisera [56] previously generated against all JEV proteins except NS2A (Figure 1A).

Of the 15 antisera, fourteen (α-C, α-Pr, α-M, α-E^N-term^, α-E^C-term^, α-NS1, α-NS1′, α-NS3^N-term^, α-NS3^C-term^, α-NS4A, α-NS4B^N-term^, α-NS4B^C-term^, α-NS5^N-term^, and α-NS5^C-term^) were produced by immunizing rabbits with recombinant GST fusion proteins expressed in *E. coli* (Figure 1B). Each fusion protein contained a non-hydrophobic region of the corresponding JEV protein fused to the C-terminus of GST. The remaining antiserum, α-NS2B, was generated using a synthetic peptide representing a defined NS2B region conjugated to KLH through the peptide’s N-terminus (Figure 1B). Together, this comprehensive panel enables high-resolution mapping of conserved and divergent antigenic regions across JEV, WNV, MVEV, and SLEV, forming the experimental foundation for the cross-reactivity analyses described below.

### 3.2. Cross-Reactivity of 15 JEV Antigen-Directed Rabbit Antisera with WNV, MVEV, and SLEV

Cross-reactivity was assessed by immunoblotting whole-cell lysates from BHK-21 cells that were either mock-infected or infected with JEV, WNV, MVEV, or SLEV at a multiplicity of infection (MOI) of 3. At 20 h post-infection, when all infected cultures exhibited similar cytopathic effects (CPE), cells were lysed in SDS-based sample buffer, boiled, separated under reducing conditions by SDS–PAGE, transferred to membranes, and probed with each of the 15 JEV antigen-directed rabbit antisera or with α-GAPDH as a loading control. Lysates from JEV-infected cells served as reference controls.

**Figure 2:**
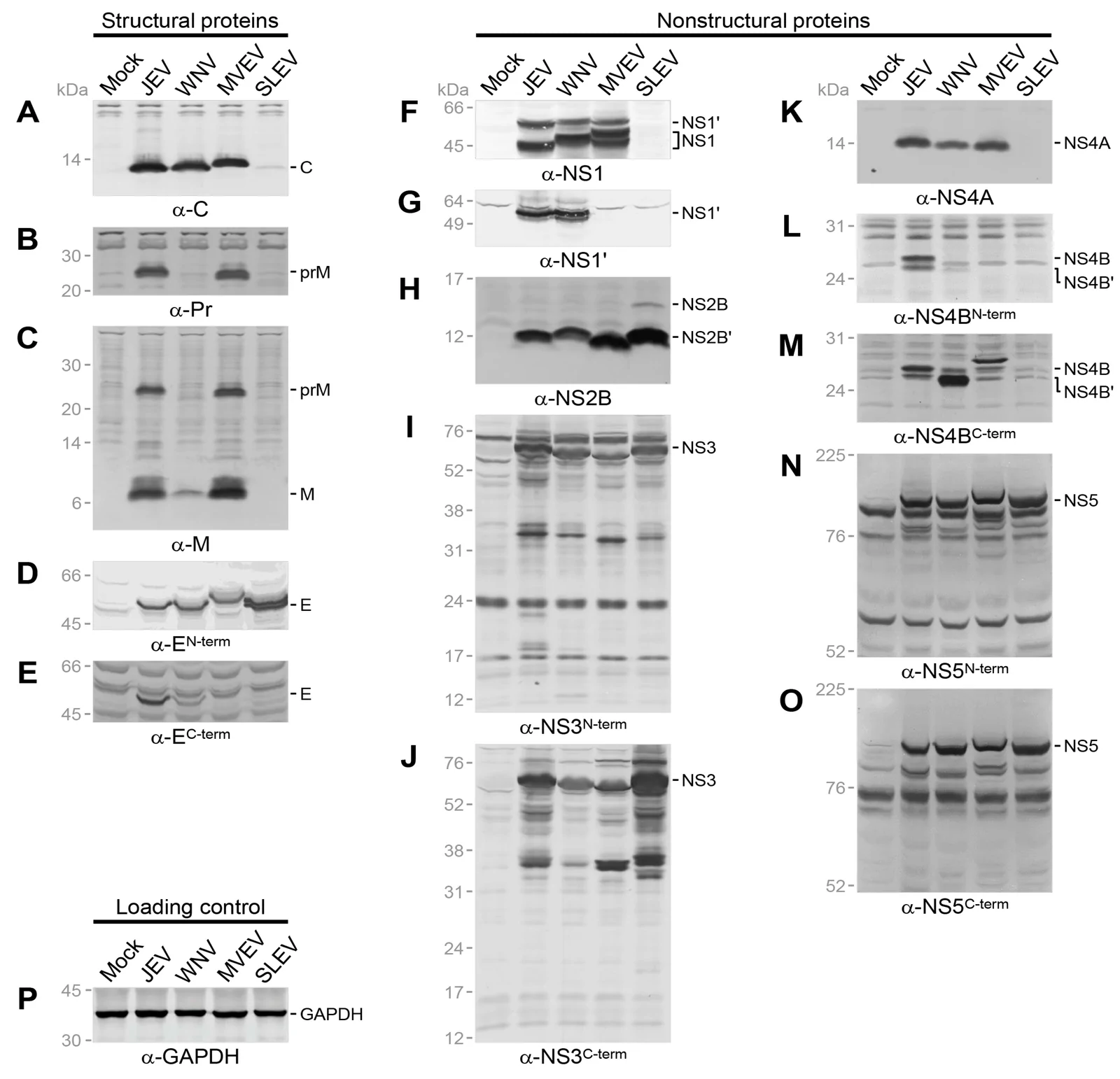
Reactivity of 15 region-specific rabbit antisera raised against JEV with four closely related encephalitic orthoflaviviruses. BHK-21 cells were mock-infected or infected with JEV (strain CNU/LP2), WNV (strain B956), MVEV (strain H102/94), or SLEV (strain H109/92) at an MOI of 3 for 20 h. Equal amounts of total cell lysates were boiled, resolved by SDS–PAGE under reducing conditions, and immunoblotted with one of the 15 JEV antigen-directed rabbit antisera: α-C (**A**), α-Pr (**B**), α-M (**C**), α-E^N-term^ (**D**), α-E^C-term^ (**E**), α-NS1 (**F**), α-NS1′ (**G**), α-NS2B (**H**), α-NS3^N-term^ (**I**), α-NS3^C-term^ (**J**), α-NS4A (**K**), α-NS4B^N-term^ (**L**), α-NS4B^C-term^ (**M**), α-NS5^N-term^ (**N**), and α-NS5^C-term^ (**O**). As a loading control, equal amounts of total cell lysates were also immunoblotted with α-GAPDH (**P**). Immunoreactive proteins were detected using AP-conjugated goat α-rabbit IgG and visualized with BCIP/NBT substrate. Predicted molecular weights for the viral proteins of all four viruses are shown as ranges, with the length of each JEV protein (in amino acids, aa) provided as a reference. Unglycosylated molecular weights are indicated for the four glycoproteins prM, E, NS1, and NS1′: C (105 aa), 11.7–11.9 kDa; prM (167 aa, unglycosylated), 18.4–19.0 kDa; M (75 aa), 8.2–8.3 kDa; E (500 aa, unglycosylated), 53.2–54.0 kDa; NS1 (352 aa, unglycosylated), 39.5–40.0 kDa; NS1′ (404 aa, unglycosylated), 45.1–45.2 kDa; NS2B (131 aa), 14.2–14.5 kDa; NS3 (619 aa), 68.5–68.9 kDa; NS4A (126 aa), 13.6–13.7 kDa; NS4B (255 aa), 27.0–27.9 kDa; NS5 (905 aa), 103.2–103.5 kDa. Predicted N-linked glycosylation sites for each glycoprotein are as follows: one site (N15) in the pr fragment of prM for all four viruses; one site (N154) in E for all four viruses; and two sites (N130 and N207) in NS1/NS1′ of JEV versus three sites (N130, N175, and N207) in NS1/NS1′ of WNV, MVEV, and SLEV.

Immunoblotting revealed distinct cross-reactivity patterns for all antisera: (*a*) α-C strongly recognized C from WNV and MVEV at levels comparable to JEV but showed markedly weaker recognition of SLEV. The MVEV C protein migrated slightly more slowly than those of the other viruses (Figure 2A). (*b*) α-Pr recognized prM from JEV and MVEV, whereas prM from WNV and SLEV was not detected under our assay conditions. The MVEV prM protein migrated slightly faster than its JEV counterpart (Figure 2B). (*c*) α-M strongly recognized prM and M from JEV and MVEV, showed relatively weak recognition of WNV M, and did not detect prM or M from SLEV (Figure 2C). (*d*) Of the two E-directed antisera, α-E^N-term^ recognized the E proteins of all four viruses. The MVEV E protein migrated slightly more slowly than those of JEV, WNV, and SLEV, and the SLEV E protein appeared as a doublet (Figure 2D). In contrast, α-E^C-term^ recognized JEV E strongly, WNV E weakly, and did not detect E from MVEV or SLEV under the conditions tested (Figure 2E). (*e*) α-NS1 strongly recognized NS1 and NS1′ from JEV, WNV, and MVEV, whereas NS1 and NS1′ from SLEV were undetectable. NS1 from WNV and MVEV migrated slightly more slowly than JEV NS1, and MVEV NS1 appeared as a doublet (Figure 2F). (*f*) α-NS1′ recognized NS1′ from JEV and WNV, while NS1′ from MVEV and SLEV was not observed under our conditions (Figure 2G). (*g*) α-NS2B reacted weakly with JEV NS2B but strongly with JEV NS2B’, and similarly recognized NS2B and NS2B’ from WNV, MVEV, and SLEV. NS2B’ from WNV and SLEV migrated slightly more slowly than JEV NS2B’, whereas MVEV NS2B’ migrated slightly faster (Figure 2H). (*h*) Both α-NS3^N-term^ and α-NS3^C-term^ strongly recognized NS3 and related species from all four viruses. NS3 from WNV and MVEV migrated slightly faster than NS3 from JEV and SLEV (Figure 2I,J). (*i*) α-NS4A recognized NS4A from JEV, WNV, and MVEV, whereas NS4A from SLEV was not detected under our assay conditions. NS4A from WNV and MVEV migrated slightly faster than JEV NS4A (Figure 2K). (*j*) Of the two NS4B-directed antisera, α-NS4B^N-term^ recognized only JEV NS4B and NS4B’, which appeared as a doublet with NS4B (upper band) more intense than NS4B’ (lower band). No NS4B-related species from WNV, MVEV, or SLEV were detected (Figure 2L). In contrast, α-NS4B^C-term^ recognized NS4B and NS4B’ from JEV, WNV, and MVEV, but not from SLEV. In WNV-infected cells, NS4B’ was more prominently detected than NS4B, and both NS4B and NS4B’ from MVEV migrated more slowly than their JEV and WNV counterparts (Figure 2M). (*k*) Both α-NS5^N-term^ and α-NS5^C-term^ strongly recognized NS5 and related species from all four viruses, with MVEV NS5 migrating slightly more slowly than the others (Figure 2N,O). Infection levels among the four viruses were comparable under our experimental conditions, as indicated by similar CPE at 20 h post-infection and by the detection of broadly reactive viral proteins (e.g., E, NS2B, NS3, and NS5) in all four infected cultures.

As summarized in Table 1, six JEV antigen-directed antisera (α-E^N-term^, α-NS2B, α-NS3^N-term^, α-NS3^C-term^, α-NS5^N-term^, and α-NS5^C-term^) displayed strong, broadly conserved cross-reactivity, consistently recognizing homologous proteins from WNV, MVEV, and SLEV. These antisera therefore detect antigenic regions that are well conserved across the four viruses. In contrast, the remaining nine antisera exhibited lineage-restricted or virus-specific recognition patterns. Specifically, α-C cross-reacted robustly with WNV and MVEV but only weakly with SLEV, indicating partial conservation of C-protein antigenic regions. Four antisera (α-M, α-NS1, α-NS4A, and α-NS4B^C-term^) recognized WNV and MVEV but not SLEV, suggesting that their antigenic regions are shared among JEV, WNV, and MVEV but diverge in SLEV. Two antisera (α-E^C-term^ and α-NS1′) cross-reacted exclusively with WNV, whereas α-Pr cross-reacted only with MVEV, highlighting antigenic features uniquely shared between JEV and either WNV or MVEV. Notably, α-NS4B^N-term^ did not detect NS4B or NS4B’ from any of the three heterologous viruses (WNV, MVEV, and SLEV) under our experimental conditions, indicating that its antigenic region is highly JEV-specific. Collectively, these findings indicate that although six of the 15 JEV antigen-directed antisera detect conserved antigenic regions across JEV, WNV, MVEV, and SLEV, the remaining nine antisera exhibit lineage-restricted or virus-specific recognition patterns, highlighting differences in antigenic determinants among these four clinically important encephalitic orthoflaviviruses.

### 3.3. Detection of Heat-Sensitive NS1- and NS1′-Associated Multimers

Orthoflavivirus NS1 is known to assemble into heat-labile multimers, including dimers, tetramers, and hexamers, through hydrophobic interactions [63,64,65,66,67,68]. To determine whether these oligomeric forms could be detected in cells infected with JEV, WNV, MVEV, or SLEV using our α-NS1 and α-NS1′ antisera, subconfluent BHK-21 cells were mock-infected or infected with each virus at an MOI of 3. At 20 h post-infection, cells were lysed in SDS-containing sample buffer, and lysates were divided so that one portion was boiled to disrupt heat-labile multimers, whereas the other remained unboiled to preserve them. Samples were analyzed under reducing conditions by SDS–PAGE, followed by immunoblotting with α-NS1 or α-NS1′.

**Figure 3:**
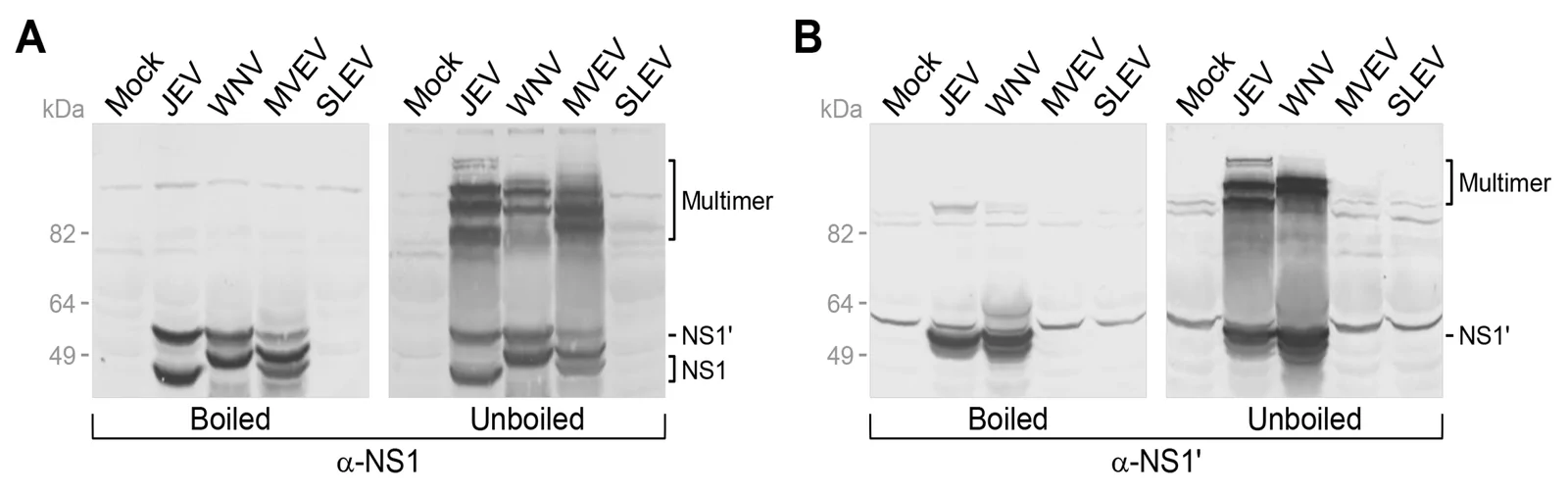
Identification of heat-unstable NS1- and NS1′-associated multimers among four closely related encephalitic orthoflaviviruses. BHK-21 cells were mock-infected or infected with JEV (strain CNU/LP2), WNV (strain B956), MVEV (strain H102/94), or SLEV (strain H109/92) at an MOI of 3 and harvested 20 h post-infection. Total cell lysates were divided and either boiled to disrupt heat-labile complexes or left unboiled to preserve them. Samples were resolved by SDS–PAGE under reducing conditions and immunoblotted with α-NS1 (**A**) or α-NS1′ (**B**). Immunoreactive proteins were detected using AP-conjugated goat α-rabbit IgG and visualized with BCIP/NBT substrate. Predicted molecular weights for monomeric unglycosylated NS1 and NS1′ are shown as ranges, with the length of each JEV protein (in amino acids, aa) provided as a reference: NS1 (352 aa), 39.5–40.0 kDa; NS1′ (404 aa), 45.1–45.2 kDa.

In boiled lysates, heat-labile NS1 and NS1′ oligomers were fully disrupted, and both antisera detected only monomeric species (Figure 3A,B, Boiled). α-NS1 recognized NS1 and NS1′ monomers in JEV-, WNV-, and MVEV-infected cells but not in SLEV-infected cells, with MVEV NS1 appearing as a doublet. α-NS1′ detected NS1′ monomers in JEV- and WNV-infected cells but not in MVEV- or SLEV-infected cells. These monomeric patterns were consistent with those observed in Figure 2F,G. In contrast, unboiled lysates preserved heat-labile oligomers. α-NS1 detected multiple higher-molecular-weight species consistent with NS1-associated multimers in JEV-, WNV-, and MVEV-infected cells but not in SLEV-infected cells (Figure 3A, Unboiled). Similarly, α-NS1′ detected several NS1′-associated multimers in JEV- and WNV-infected cells but not in MVEV- or SLEV-infected cells (Figure 3B, Unboiled). Each virus exhibited a distinct pattern of immunoreactive bands, indicating virus-specific differences in the abundance and size distribution of NS1/NS1′ multimers. No NS1- or NS1′-associated oligomers were detected in mock-infected controls. Together, these findings demonstrate that α-NS1 detects NS1- and NS1′-associated multimers in JEV-, WNV-, and MVEV-infected cells, whereas α-NS1′ detects NS1′-associated multimers only in JEV- and WNV-infected cells. This differential recognition highlights virus-specific variation in NS1/NS1′ oligomerization and underscores the utility of these antisera for probing NS1/NS1′ structural dynamics.

### 3.4. Sequence Comparison of the −1 Frameshift-Stimulating Signal Required for NS1′ Expression

**Figure 4:**
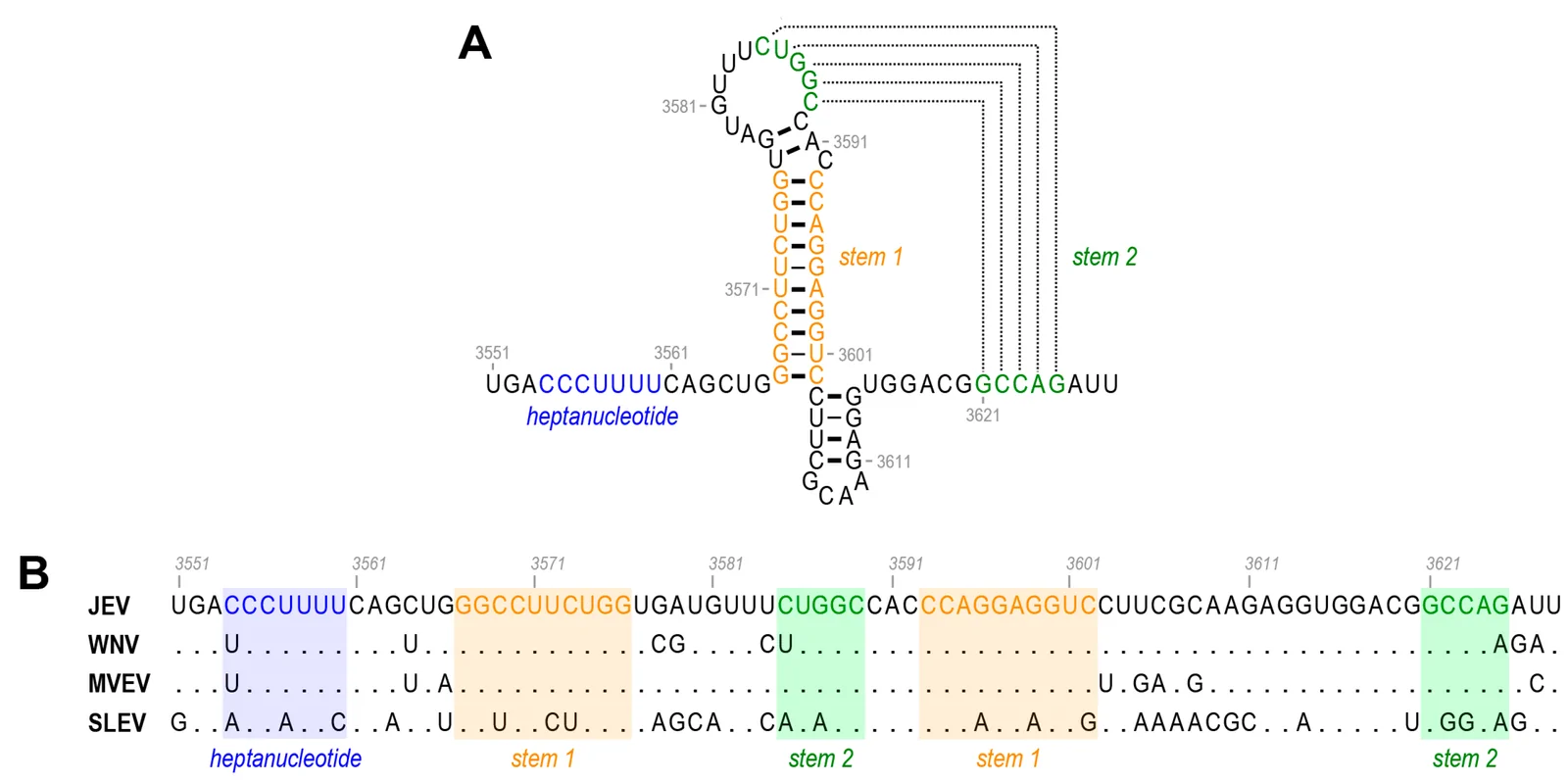
Programmed ribosomal frameshift signal directing NS1′ expression in JEV and its conservation among four closely related encephalitic orthoflaviviruses. (**A**) Primary nucleotide sequence and predicted secondary structure spanning nucleotides 3551–3628 of the genomic RNA of JEV strain CNU/LP2 (GenBank AY585243), illustrating the cis-acting elements required for −1 ribosomal frameshifting. The predicted pseudoknot, located immediately downstream of the slippery heptanucleotide, is shown with its two constituent stems, both of which are essential for stimulating the frameshift event. (**B**) Comparative nucleotide sequence alignment of the corresponding genomic region from JEV strain CNU/LP2 with three additional JEV-serogroup viruses: WNV strain B956 (GenBank AY532665), MVEV strain MVE-1-51 (GenBank AF161266), and SLEV strain Imperial Valley (GenBank JF460774). The slippery heptanucleotide (blue) and the two pseudoknot stems, stem 1 (orange) and stem 2 (green), are highlighted to emphasize conserved and divergent structural features. Nucleotide positions correspond to the numbering of the JEV CNU/LP2 genome.

NS1′ is known to be expressed in JEV and other JEV-serogroup viruses through a programmed −1 ribosomal frameshift that occurs at codons 8–9 of NS2A [42,43,69,70]. This frameshift is thought to be driven by two cis-acting RNA elements: a slippery heptanucleotide sequence and a downstream stimulatory pseudoknot (Figure 4). In the consensus model, the slippery motif (YCCUUUU, where Y is C or U) lies immediately upstream of a two-stem pseudoknot, in which stem 2 is formed by base-pairing between the loop of the stem-1 hairpin and a complementary downstream region [42].

To assess conservation of this frameshift signal, we compared nucleotides 3551–3628, the region implicated in −1 ribosomal frameshifting, across JEV, WNV, MVEV, and SLEV, using the well-characterized JEV sequence as the reference [69,70]. JEV contains the canonical slippery sequence (3554-CCCUUUU-3560) and a typical two-stem pseudoknot, in which nucleotides 3585–3589 (CUGGC) within the loop atop stem 1 base-pair with nucleotides 3621–3625 (GCCAG) to form stem 2 (Figure 4A). WNV and MVEV both retain a motif-compatible slippery sequence (UCCUUUU) and preserve the overall pseudoknot architecture. Their stem-1 and stem-2 sequences are identical to those of JEV, except that WNV carries compensatory substitutions at positions 3585 (C→U) and 3625 (G→A). These substitutions replace the C:G base pair with a U:A base pair in stem 2 while maintaining the predicted pseudoknot structure (Figure 4B). In contrast, SLEV lacks both a recognizable slippery heptamer and a two-stem pseudoknot within the corresponding genomic region (Figure 4B). No alternative structures consistent with a −1 frameshift-stimulating signal were apparent. Together, these findings indicate that the −1 frameshift signal required for NS1′ expression is conserved in the genomes of JEV, WNV, and MVEV but is not evident in the genome of SLEV.

### 3.5. Phylogenetic Analysis of JEV Serogroup Members Within the Genus Orthoflavivirus

A phylogenetic analysis was performed to compare the serological relationships observed among JEV, WNV, MVEV, and SLEV with the corresponding genetic relationships of their genomes. Complete or near-complete genome sequences from all eight established members of the JEV serogroup, together with 38 additional orthoflaviviruses representing the diversity of the genus, were included. BVDV, a pestivirus within the *Flaviviridae* family, served as the outgroup.

**Figure 5:**
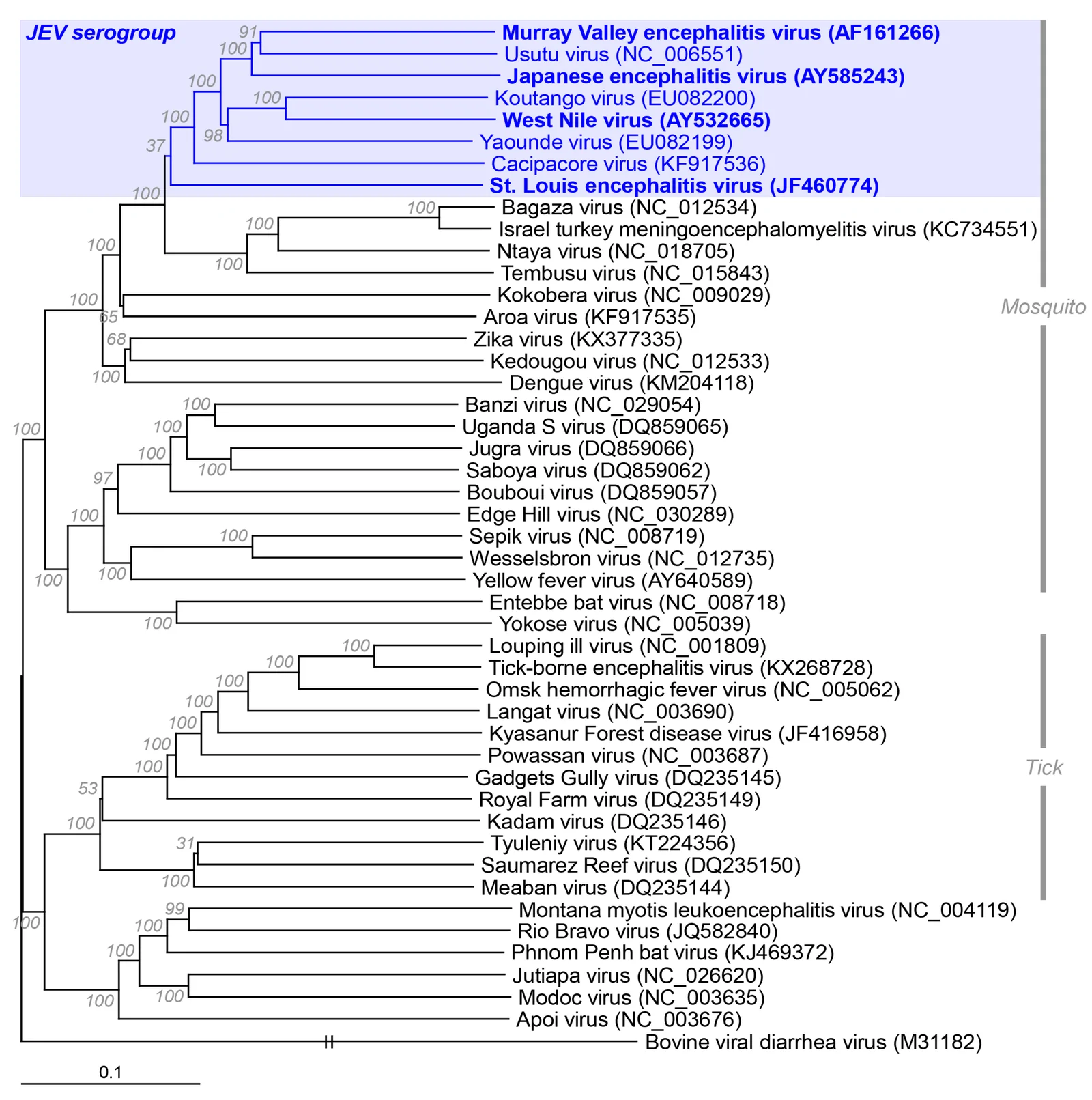
Genome-based phylogeny of 46 orthoflaviviruses highlighting the JEV serogroup. The rooted phylogenetic tree was constructed using 1000 bootstrap iterations, with bovine viral diarrhea virus serving as the outgroup. Bootstrap values are shown as percentages. Mosquito-borne and tick-borne orthoflaviviruses are grouped and labeled. Members of the JEV serogroup are highlighted in blue. The scale bar represents evolutionary distance in nucleotide substitutions per site. Nucleotide sequences were obtained from GenBank, with accession numbers provided in parentheses.

As shown in Figure 5, the rooted phylogeny revealed that all eight JEV-serogroup viruses, including the four widely studied members (JEV, WNV, MVEV, and SLEV) and four lesser-known members (USUV, KOUV, YAOV, and CPCV), formed a monophyletic cluster within the mosquito-borne clade. This cluster was clearly distinct from other mosquito-borne orthoflavivirus lineages, including those represented by the Aroa, dengue, Kokobera, Ntaya, and yellow fever virus serogroups, as well as the Spondweni virus serogroup, whose representative in the phylogeny was the Zika virus, the closest known relative of the Spondweni virus. Within the JEV serogroup, SLEV consistently emerged as the earliest-diverging lineage, indicating a deeper evolutionary separation from the remaining members. CPCV branched next, followed by two strongly supported subclusters: one comprising JEV, MVEV, and USUV, and the other comprising WNV, KOUV, and YAOV. These two subclusters formed sister clades, together defining the internal structure of the JEV serogroup. Overall, the phylogeny highlights both the distinct placement of the JEV serogroup within the *Orthoflavivirus* genus and the structured evolutionary relationships among its members. Notably, the early phylogenetic divergence of SLEV and the close clustering of JEV, MVEV, and WNV parallel the immunoblot-based serological patterns observed in this study, supporting general consistency between antigenic detection and genetic relatedness.

## 4. Discussion

Although members of the JEV serogroup are genetically closely related, their antigenic relationships remain incompletely defined [1,2]. This gap continues to hinder progress in serological diagnostics [71,72,73,74], vaccine development [75,76,77,78], and comparative pathogenesis research [3,4,79]. By systematically profiling the cross-reactivity of 15 region-specific rabbit antisera raised against nearly all JEV proteins, we characterized how individual viral proteins contribute to patterns of antigenic similarity or differences in antibody recognition among four major serogroup members: JEV, WNV, MVEV, and SLEV. The resulting reactivity patterns were highly structured and broadly aligned with phylogenetic relationships. Six antisera (α-E^N-term^, α-NS2B, α-NS3^N-term^, α-NS3^C-term^, α-NS5^N-term^, and α-NS5^C-term^) recognized homologous proteins across all four viruses, indicating that the antigenic regions they detect are conserved. In contrast, the remaining nine antisera revealed lineage-restricted or virus-specific recognition patterns: α-C reacted strongly with JEV, WNV, and MVEV but only weakly with SLEV; α-M, α-NS1, α-NS4A, and α-NS4B^C-term^ recognized JEV, WNV, and MVEV but not SLEV; α-E^C-term^ and α-NS1′ recognized only JEV and WNV; α-Pr recognized only JEV and MVEV; and α-NS4B^N-term^ was specific to JEV. These distinct recognition profiles, including differential detection of heat-labile NS1 and NS1′ multimers, mirror the evolutionary structure of the serogroup and demonstrate the utility of this antiserum panel as a serological toolkit for distinguishing closely related encephalitic orthoflaviviruses.

Serogroup classification within the genus *Orthoflavivirus* has traditionally relied on serological reactivity to the viral surface E protein, based on the expectation that viruses within the same serogroup exhibit cross-reactivity to their E proteins [3,4,71,79,80]. Although this framework has guided diagnostic and taxonomic practice for decades, the specific regions of E responsible for cross-reactivity have remained insufficiently defined. In our study, the α-E^N-term^ antiserum, raised against the N-terminal 116-aa region of JEV E, containing four β-strands (*A*_0_, *B*_0_, *C*_0_, and *D*_0_) of Domain I and three β-strands (*a*, *b*, and *c*) plus the fusion loop of Domain II [81], recognized the E proteins of all four viruses. This broad recognition indicates that the antigenic region detected by α-E^N-term^ is serologically indistinguishable by immunoblotting among JEV, WNV, MVEV, and SLEV and likely represents part of the conserved antigenic core historically associated with serogroup definition. In contrast, the α-E^C-term^ antiserum, directed against an internal 102-aa region of JEV E that includes two β-strands (I_0_ and J_0_) of Domain I and two α-helices (αA and αB) plus six β-strands (*g*, *h*, *i*, *j*, *k*, and *l*) of Domain II [81], recognized JEV strongly, WNV weakly, and did not detect E from MVEV or SLEV under the conditions tested. This pattern suggests that, unlike the α-E^N-term^-recognized region, the α-E^C-term^-recognized region captures antigenic differences that distinguish JEV from WNV and clearly separate JEV from MVEV and SLEV. These findings refine the classical serogroup concept by demonstrating that distinct subregions of E encode different degrees of antigenic similarity or differences in antibody recognition.

Several antisera recognized all four JEV serogroup viruses, expanding the antigenic landscape beyond the traditional focus on the E protein. The α-C antiserum, raised against a nearly full-length JEV C protein containing four α-helices but lacking the C-terminal transmembrane domain [82], recognized C from all four viruses, although recognition of SLEV was noticeably weaker. This pattern suggests that the C protein contains conserved antigenic regions, but that differences in SLEV reduce antibody recognition under the conditions tested. Antisera targeting the N-terminal or C-terminal halves of NS3 [83,84] and NS5 [85], as well as an internal 12-aa region of NS2B that interacts with NS3 as a protease cofactor [86,87,88], also recognized their corresponding proteins across all four viruses. These findings indicate that NS2B, NS3, and NS5, three of the most genetically conserved orthoflaviviral proteins [89,90,91], are also serologically indistinguishable, reinforcing the idea that conserved nonstructural proteins encode deeply shared antigenic determinants. Additional observations further support this interpretation: α-NS2B strongly recognized NS2B’ from DENV but not YFV (unpublished), and α-NS5^C-term^ detected NS5 from both DENV and YFV with relatively weak reactivity (unpublished). These results suggest that certain antigenic regions within NS2B and NS5 may be conserved not only within the JEV serogroup but also across more distantly related orthoflaviviruses. Thus, among our 15 JEV antigen-directed rabbit antisera, α-NS2B and α-NS5^C-term^ stand out as reagents capable of recognizing their corresponding proteins across all four major JEV serogroup viruses and potentially additional non-JEV-serogroup orthoflaviviruses, including DENV and YFV. This cross-reactivity highlights the evolutionary stability of these nonstructural proteins and their potential utility as broadly reactive serological tools.

From an evolutionary standpoint, our serological findings support the interpretation that JEV is most closely related to MVEV, followed by WNV and then SLEV, consistent with established phylogenetic data [6,7]. When the five antisera targeting highly conserved nonstructural proteins (α-NS2B, α-NS3^N-term^, α-NS3^C-term^, α-NS5^N-term^, and α-NS5^C-term^) are excluded, a clearer hierarchy of antigenic relatedness emerges. First, JEV and MVEV exhibited the strongest antigenic resemblance: all MVEV structural proteins were robustly detected by α-C, α-Pr, α-M, and α-E^N-term^. Notably, α-Pr, raised against the entire pr region of JEV prM, recognized prM from JEV and MVEV but not WNV or SLEV, highlighting a distinctive antigenic link between these two viruses. Three antisera targeting nonstructural proteins (α-NS1, α-NS4A, and α-NS4B^C-term^) also reacted with MVEV, further supporting their close relationship. Second, WNV displayed an intermediate level of relatedness: all WNV structural proteins were detected by α-C, α-M, α-E^N-term^, and α-E^C-term^, although α-M and α-E^C-term^ showed minimal reactivity. The absence of α-Pr reactivity underscores the antigenic distinction between WNV and the more closely related JEV and MVEV. WNV was also recognized by α-NS1, α-NS4A, and α-NS4B^C-term^, similar to MVEV, and uniquely by α-NS1′. Third, SLEV showed the greatest differences in antibody recognition: only α-E^N-term^ reacted strongly with the E protein, and α-C bound only weakly to the C protein. Of the two NS4B-directed antisera, α-NS4B^N-term^ (targeting a 40-aa region near the N-terminus) was specific to JEV, whereas α-NS4B^C-term^ (targeting a 60-aa region near the C-terminus) cross-reacted with JEV, WNV, and MVEV but not SLEV. Together, these serological profiles delineate antigenic boundaries within the JEV serogroup and complement sequence-based phylogenetic relationships.

Orthoflavivirus NS1 is known to form heat-labile homo-oligomers that underpin its diverse biological functions [63,64,65,66,67,68]. The intracellular homodimer supports viral RNA replication, whereas the secreted homotetramer and homohexamer contribute to viral pathogenesis, facilitate immune evasion, and serve as diagnostic markers [40,45,92,93,94]. These multimers illustrate the structural versatility of NS1 and demonstrate how a single viral protein participates in both intracellular replication and extracellular immune modulation [44,95]. Members of the JEV serogroup also produce NS1′ through a programmed −1 ribosomal frameshift at codons 8–9 of NS2A, adding 52 amino acids to the C-terminus of NS1 [42,43,69,70]. Although the oligomeric states of NS1′ have not been experimentally defined, it is reasonable to assume that NS1′ adopts multimeric forms similar to NS1, given that the primary interaction domains reside within the NS1 portion of the protein, particularly the N-terminal β-roll domain of approximately 30 amino acids [63]. Previous studies have reported NS1′ expression in JEV, WNV, and MVEV [96,97,98,99,100], and our findings are consistent with these observations. Specifically, α-NS1, which targets the N-terminal 166-aa region shared by NS1 and NS1′, recognized both proteins from JEV, WNV, and MVEV. In contrast, α-NS1′, which targets the C-terminal 43-aa region unique to NS1′, recognized NS1′ from JEV and WNV but not MVEV. The absence of a detectable NS1′ signal for MVEV likely reflects differences in antigenic determinants that limit recognition by α-NS1′, as MVEV NS1′ was readily detected by α-NS1. Moreover, both α-NS1 and α-NS1′ antisera detected monomers and heat-labile multimers, although whether these multimers include NS1–NS1′ hetero-oligomers remains unknown. We also identified a canonical frameshift signal conserved in nucleotides 3551–3628 of the JEV genome and in the corresponding regions of WNV and MVEV, but not SLEV. The absence of this signal in SLEV aligns with earlier reports suggesting that SLEV may rely on a non-canonical frameshift site, potentially located within the NS1 coding region upstream of the site used by JEV, WNV, and MVEV [42]. The absence of a detectable NS1′ signal for SLEV may reflect either a true lack of NS1′ expression or differences in antigenic determinants that limit recognition by α-NS1′. This raises the possibility that NS1′ expression in SLEV may be regulated differently or may not occur at all, highlighting an important gap in our understanding of SLEV biology. The presence and functional relevance of NS1′ in SLEV therefore remain to be experimentally validated.

Several limitations should be considered when interpreting our findings. Immunoblotting captures linear antigenic regions and may not fully represent conformational determinants, which could broaden the apparent cross-reactivity profiles of the antisera tested. In addition, only one strain each of JEV, WNV, MVEV, and SLEV was examined, and strain-specific variation could influence the observed reactivity patterns. Future work should expand this approach to additional orthoflaviviruses to further clarify antigenic relationships within the JEV serogroup and across the *Orthoflavivirus* genus. Mapping conserved and variable antigenic regions will also support the development of more specific diagnostic assays, an important need in regions where multiple orthoflaviviruses co-circulate and serological overlap complicates surveillance and clinical decision-making.

In summary, this study provides the first comprehensive genome-wide serological analysis across four clinically important, phylogenetically related neurotropic orthoflaviviruses: JEV, WNV, MVEV, and SLEV. By integrating protein-level expression data with region-specific serological profiling, we offer a refined view of antigenic similarities and differences within the JEV serogroup. These insights may support improved diagnostic specificity, inform rational vaccine design, and guide the development of broadly protective immunogens, representing potential future applications to strengthen preparedness for emerging and re-emerging orthoflaviviruses.

## Figures and Tables

**Table 1:** Summary of the reactivity of 15 JEV antigen-directed rabbit antisera with four representative members of the JEV serogroup.

		Reactivity (Relative Intensity) ^1^			
Antiserum	Major Proteins Detected	JEV ^2^	WNV	MVEV	SLEV
α-C	C	Strong (1.0)	Strong (1.0)	Strong (0.9)	Weak (0.1)
α-Pr	prM	Strong (1.0)	―	Strong (1.0)	―
α-M	prM and M	Strong (1.0)	Weak (0.2)	Strong (1.2)	―
α-E^N-term^	E	Strong (1.0)	Strong (1.2)	Strong (1.1)	Strong (2.1)
α-E^C-term^	E	Strong (1.0)	Weak (0.3)	―	―
α-NS1	NS1 and NS1′	Strong (1.0)	Strong (1.2)	Strong (1.3)	―
α-NS1′	NS1′	Strong (1.0)	Strong (1.1)	―	―
α-NS2B	NS2B and NS2B’	Strong (1.0)	Strong (1.1)	Strong (1.4)	Strong (1.7)
α-NS3^N-term^	NS3 and related proteins	Strong (1.0)	Strong (0.7)	Strong (0.5)	Strong (0.8)
α-NS3^C-term^	NS3 and related proteins	Strong (1.0)	Strong (0.5)	Strong (0.7)	Strong (1.8)
α-NS4A	NS4A	Strong (1.0)	Strong (0.7)	Strong (0.9)	―
α-NS4B^N-term^	NS4B and NS4B’	Strong (1.0)	―	―	―
α-NS4B^C-term^	NS4B and NS4B’	Strong (1.0)	Strong (1.8)	Strong (1.0)	―
α-NS5^N-term^	NS5 and related proteins	Strong (1.0)	Strong (1.0)	Strong (1.0)	Strong (1.3)
α-NS5^C-term^	NS5 and related proteins	Strong (1.0)	Strong (1.5)	Strong (1.2)	Strong (1.6)

^1^ Reactivity of the 15 JEV antigen-directed rabbit antisera with JEV, WNV, MVEV, and SLEV was evaluated by immunoblot analysis. Reactivity was classified as “Strong” when the immunoreactive band intensity was more than 50% of that observed for JEV and “Weak” when the intensity was less than 50% of that observed for JEV. The symbol “―” indicates no detectable reactivity under the conditions tested. Major viral protein band intensities were quantified by densitometric analysis using ImageJ. For each sample, the intensity of each major viral-protein band was divided by the intensity of the corresponding GAPDH band to obtain a viral-protein/GAPDH ratio. The resulting ratios were then normalized to the JEV control, which was assigned a value of 1.0. Normalized densitometry values are shown in parentheses. ^2^ Viral strains analyzed in this study were JEV CNU/LP2, WNV B956, MVEV H102/94, and SLEV H109/92.

## Data Availability

All data generated in this study are included within this manuscript or are available from the authors upon request.
